# Diversifying cheminformatics

**DOI:** 10.1186/s13321-022-00597-5

**Published:** 2022-04-25

**Authors:** Barbara Zdrazil, Rajarshi Guha

**Affiliations:** 1 Department of Pharmaceutical Sciences, University of Vienna, Althanstraße 14, 1090 Vienna, Austria; 2 Vertex Pharmaceuticals, 50 Northern Ave, Boston, MA 02210 USA

With Dr. Barbara Zdrazil starting in her role as Co-Editor-in-Chief in January 2022, we revisit the scope of *J. Cheminform*, as well as the role of cheminformatics as a discipline. We present our vision for the Journal in moving the field of cheminformatics as well as Open Science forward in the coming years.

## Cheminformatics as a bridging discipline

By joining the fields of chemistry and information technology for solving chemical problems related to storage, indexing, and searching of chemical information, a tool was created to solve complex problems in drug discovery [1]. While chem- and bioinformatics are key to drug discovery workflows, cheminformatics is an important tool in disciplines beyond drug discovery, such as materials science [2], metabolomics [3], and odor research [4]. We believe that *J. Cheminform.* plays a key role within the cheminformatics community as a platform to disseminate descriptions (and implementations) of cheminformatics methods. This belief drives our focus on research reproducibility, requiring data and code being made openly available to the public, building an important foundation in order to accelerate basic research.

Therefore, over the next 4 years we will focus our editorial efforts on these three main topics:

- Improving research reproducibility, open access data and code;
- Publishing benchmark studies for machine learning and artificial intelligence-based studies to better understand the utility of different algorithms;
- Expanding our support of diversity in cheminformatics: both from a topical aspect, where we highlight work in interdisciplinary and niche areas; and from a community aspect, where we increase the visibility of underrepresented groups and regions.

## Improving research reproducibility

As we continue to follow previously defined publishing practices of the journal regarding re-usable and fully accessible content of the journal articles (including published software, data, and algorithms), we understand that there will be further efforts needed to better define reproducibility in a cheminformatics and computational chemistry setting. As R. Clark recently pointed out, there is no simple way of validating your algorithm, since it will always give the same results when applied to the same data set and under the same conditions (unlike in an experimental setting) [5]. While a rigorous re-implementation of algorithms as suggested by Clark is out of reviewers’ capacities, we are now starting an effort to engage in more active code reviews during the paper review processes in addition to enquiring on the availability of source code and data for reproducing the results of the paper.

The other aspect of improving research reproducibility is the encouragement of standardized formats for data submissions. One such effort that we intend to adopt and expand is based on work from Schymanski and Bolton [6], which will encourage authors to submit their chemical data via a chemical structure template and thereby link the DOI of the data file to the article DOI metadata. While we do not currently mandate such a submission format, as Editors, we will encourage authors and work with them to apply this template where possible.

## Machine learning & cheminformatics

**Fig. 1:**
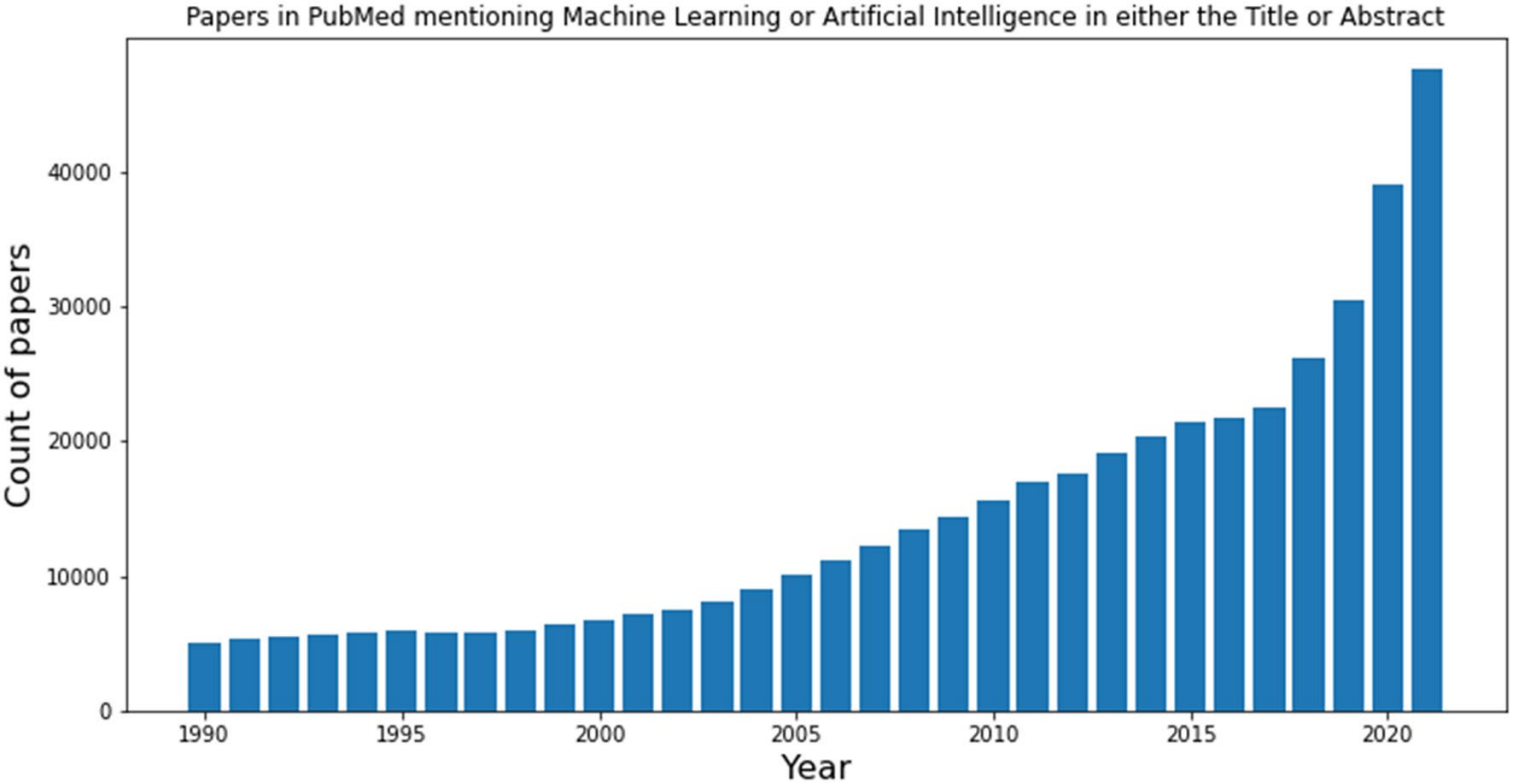
Time trend showing the number of published papers in PubMed from 1990–2021 mentioning “Machine Learning”, “Artificial Intelligence”, “ML” or “AI” in either paper title or abstract

The last decade has seen a tremendous rise in the use of machine learning (ML) algorithms across natural sciences. A bibliometric analysis highlights how the number of yearly published papers in the domain of ML has increased over time (Fig. 1). With the rise in new methods and applications, we feel that it is a necessary and timely undertaking to critically revise the numerous algorithms and assemble information about strengths and limitations of the various methods. One way to do so is to invite submissions that report on benchmarking studies. Recently, there has been some discussion around defining standards to enable rigorous comparisons of this kind [7, 8] , which of course also includes the discussion about the appropriate statistical tests for the use cases at hand. As we believe those are important discussions to have community-wide in order to bring our field forward and make it fit for the next generation of ML data scientists, we will foster initiatives in these directions in the near future. This will include publishing thematic issues on the topics of benchmarking studies for ML, statistical validation, etc.

## Diversifying cheminformatics

Our third focus, on diversity, aims to increase the breadth of topics we publish on and the breadth of authors that may be working in this area.

*J. Cheminform. *is not just about publishing manuscripts, but also serves as a means to coordinate and encourage a multitude of research topics and researchers. The journal’s success opens up a great opportunity for us to aid the transition of the current research culture into a more divers and open-minded environment. While broadening the scope of cheminformatics papers will be undertaken by dedicated thematic collections, which will also include niche topics, we hope to address the latter by broadening representation on our editorial board, developing thematic issues that focus on careers in this field, and in particular, highlighting challenges and influences that scientists from different underrepresented groups are facing.

## Conclusion

Our aim for the next four years is to bring open science efforts together with strategic plans to broaden the scope of the journal to be more diverse and inclusive while fostering initiatives to provide a platform for timely discussions around artificial intelligence-based algorithms and studies. The authors, reviewers and Editorial Board of *J. Cheminform.* have made great contributions to increase the quality of the articles published by the journal in the past, and we would like to acknowledge this community effort. Diversifying cheminformatics will only be possible with continued contributions by our community, in terms of submitting articles as well as active engagement in discussing the future role of cheminformatics.
